# Resveratrol Prevents *Campylobacter jejuni*-Induced Leaky gut by Restoring Occludin and Claudin-5 in the Paracellular Leak Pathway

**DOI:** 10.3389/fphar.2021.640572

**Published:** 2021-04-15

**Authors:** F. D. Lobo de Sá, M. M. Heimesaat, S. Bereswill, P. K. Nattramilarasu, J. D. Schulzke, R. Bücker

**Affiliations:** ^1^ Medical Department, Division of Gastroenterology, Infectious Diseases, Rheumatology, Nutritional Medicine/Clinical Physiology, Charité–Universitätsmedizin Berlin, Berlin, Germany; ^2^ Institute of Microbiology, Infectious Diseases, and Immunology, Gastrointestinal Microbiology Research Group, Charité - Universitätsmedizin Berlin, Berlin, Germany

**Keywords:** epithelial barrier, mucosal permeability, leak pathway, tight junction, epithelial apoptosis, leak flux, leaky gut model

## Abstract

*Campylobacter jejuni* is a bacterial human pathogen causing gastroenteritis and sequelae like irritable bowel syndrome. Epidemiologists count the human campylobacteriosis by *C. jejuni* as the most common foodborne zoonosis and bacterial diarrheal disease worldwide. Based on bioinformatics predictions for potential protective compounds in campylobacteriosis, the question was raised whether the plant-based polyphenol resveratrol is sufficient to attenuate intestinal epithelial damage induced by *C. jejuni*. We investigated this by performing experimental infection studies in an epithelial cell culture and the secondary abiotic IL-10^−/−^ mouse model. In *C. jejuni*-infected human colonic HT-29/B6 cell monolayers, transepithelial electrical resistance (TER) was decreased and the paracellular marker flux of fluorescein (332 Da) increased. Concomitantly, the tight junction (TJ) proteins occludin and claudin-5 were re-distributed off the tight junction domain. This was accompanied by an increased induction of epithelial apoptosis, both changes contributing to compromised barrier function and the opening of the leak pathway induced by *C. jejuni*. In parallel, the recovery experiments with the application of resveratrol revealed a functional improvement of the disturbed epithelial barrier in both models *in vitro* and *in vivo*. During treatment with resveratrol, TJ localization of occludin and claudin-5 was fully restored in the paracellular domain of HT-29/B6 cells. Moreover, resveratrol decreased the rate of epithelial apoptosis. These resveratrol-induced molecular and cellular effects would therefore be expected to improve epithelial barrier function, thereby minimizing the so-called leaky gut phenomenon. In conclusion, the induction of the leak pathway by *C. jejuni* and the restoration of barrier function by resveratrol demonstrates its effectiveness as a potential preventive or therapeutic method of mitigating the leaky gut associated with campylobacteriosis.

## Introduction

*Campylobacter jejuni* is a Gram-negative bacterial human pathogen of the gastrointestinal tract. It is a microaerophilic, motile, curved-shaped rod, belonging to the epsilon-proteobacteria group, related to the genera *Helicobacter*, *Wolinella* and *Aliarcobacter* (Vandamme et al., 1991). In animal hosts, mainly in the intestines of domestic and wild poultry, the bacteria are commensals (Shane, 1992). Transmission of *C. jejuni* to humans occurs mostly via undercooked meat or contaminated food (Shane, 1992). After oral uptake of *C. jejuni* by the human host, the bacteria penetrate the intestinal mucus and invade the epithelium in the small and large intestine, leading to diarrhea, gastroenteritis, and fever (Wassenaar and Blaser, 1999).

*Campylobacter jejuni* is able to actively migrate through the intestinal epithelial layer and thereby causes dysfunction of the epithelial barrier, inducing diarrhea by the *leak flux* mechanism (Bücker et al., 2018; Butkevych et al., 2020). Hundreds of attempts to find an exotoxin from *C. jejuni* that could explain its barrier-breaking effects were not successful. The missing evidence for an exotoxinogenic barrier leakage by the bacteria led to the conclusion that the lipooligosaccharides (LOS) and other endotoxins of the bacteria mediate the main pathophysiological events of barrier dysfunction and an exuberant immune response in the mucosal layers (Wassenaar and Blaser, 1999). The major role of LOS in *C. jejuni*-mediated intestinal pathogenesis was further supported recently by independent findings in humans (Bücker et al., 2018) and in various murine models of campylobacteriosis (reviewed by Mousavi et al., 2020). The downward spiral in the infected intestinal mucosa includes the innate immune activation including subepithelial macrophages and granulocytes (a *mucosal cytokine storm*), which in turn aggravates epithelial leakage, which leads again to further influx of antigens (Butkevych et al., 2020; Lobo de Sá et al., 2021). The so-called *leaky gut* phenomenon is the model representation for this cytokine-induced barrier impairment (Hollander, 1999; Schulzke et al., 2006). To describe the pathogenesis of this phenomenon, intestinal biopsies from animals or patients as well as cell culture monolayers interacting with subepithelial cytokine release from lymphocytes, can be used as experimental leaky gut models (Bücker et al., 2014; Bücker et al., 2018).

However, the molecular and structural changes in the *C. jejuni*-infected epithelium include induction of epithelial apoptosis and downregulation of tight junction (TJ) protein expression by genomic regulation of claudin expression, accompanied by a subcellular redistribution of barrier-forming claudins. The direct (bacterial) and indirect (inflammatory) pathological events caused by *C. jejuni* in the epithelium explain infective/inflammatory diarrhea in humans. Pro-inflammatory activation is the hallmark of infection (Black et al., 1988), in which the release of barrier-affecting cytokines, such as tumor necrosis factor alpha (TNF-α), interferon gamma (IFN-γ), or interleukin-1 beta (IL-1β) within the intestinal mucosa seems to play a major role in the perpetuation of the diarrhea (Rees et al., 2008; Amasheh et al., 2010; Luettig et al., 2016). Pro-inflammatory cytokines and the bacterium *per se* target epithelial TJ proteins and trigger epithelial cell death mechanisms (Bücker et al., 2018).

Curcumin from the Turmeric root, *Curcuma longa* L., is effective against *C. jejuni* infection *in vitro* as well as *in vivo* (Lobo de Sá et al., 2019). Resveratrol is another polyphenolic compound and traditional remedy, which we have shown in the present study to possess barrier-restorative properties in different models. In the literature, plant-derived traditional remedies containing resveratrol are broadly described for treatment options of different diseases including inflammatory or cardiovascular diseases (Chudzińska et al., 2020).

The polyphenolic micronutrient resveratrol (3, 5, 4′-trihydroxystilbene) is a bioactive compound found in various plants such as wine grapes (0.1–3.0 μg/g), berries (∼0.03 μg/g), peanuts (0.01–2.00 μg/g), and in the traditional Asian remedy for inflammatory indications, the Japanese knotweed *Reynoutria japonica*, which has the highest concentration of resveratrol (>500 μg/g) (Koushki et al., 2018a; Liu et al., 2020). For the preparation of knotweed, recipes from Japan or Korea and in traditional Chinese medicine recommended the use of young raw sprouts as salad or the roots as tincture or powder, to provide sufficient pharmacological concentrations of resveratrol. The anti-inflammatory properties of resveratrol were described in several studies. Improvement of the intestinal epithelial barrier by the molecular effects of resveratrol on TJ stability under inflammatory conditions independently of *C. jejuni* infection have also been reported (Carrasco-Pozo et al., 2013; Ling et al., 2016; Mayangsari and Suzuki, 2018; Cao et al., 2019). The phytopharmaceutical resveratrol is effective in reducing inflammatory parameters such as TNF-α or C-reactive protein (CRP) levels in clinical trials (Koushki et al., 2018b). Effective concentrations of resveratrol are not usually provided by a normal diet (Weiskirchen and Weiskirchen, 2016). Only traditional remedy plants or pharmaceutical preparations provide pharmacological relevant concentrations of resveratrol, but these can cause adverse effects (Weiskirchen and Weiskirchen, 2016; Salehi et al., 2018).

Concerning *C. jejuni* infections, we have shown recently that resveratrol treatment alleviates intestinal inflammation and macroscopic sequelae of campylobacteriosis in a standardized murine preclinical disease model (Heimesaat et al., 2020). To determine mechanisms underlying the beneficial effects of resveratrol in the treatment of campylobacteriosis, we performed experiments in different models. Human intestinal HT-29/B6 cell monolayers in conjunction with inflammatory conditions were used as *in vitro* model of the leaky gut, and an *in vivo* hyperacute animal model was used to study intestinal leakiness and inflammation. The *Campylobacter*-infected secondary abiotic IL-10^−/−^ mouse model was used to confirm the mechanistic effects observed *in vitro*.

## Materials and Methods

### Bioinformatics on RNA-Seq Data

Based on a previously performed RNA-Sequencing (RNA-Seq) analysis with subsequent Ingenuity Pathway Analysis (IPA; Qiagen, Redwood, CA, United States) of human colonic mucosa from four *C. jejuni* infected patients and six control patients, several compounds were identified as potential inhibitory or therapeutic agents in campylobacteriosis. Procedures of RNA extraction, RNA sequencing, and Ingenuity Pathway Analysis were performed previously (Bücker et al., 2018). The sequence data set for the bioinformatics prediction is available under NCBI Gene Expression Omnibus (GEO ID GSE88710). In the IPA prediction for the potential compound resveratrol, the Upstream regulator analysis software tool was used. The detailed information on the calculation method and statistics is available on Ingenuity–Qiagen websites. In brief, Upstream regulator analysis is based on prior knowledge from the literature of expected effects between transcriptional regulators and their target genes. The analysis examines how many known targets of each transcription regulator are regulated in the RNA dataset of the patients. For each potential transcriptional regulator, two statistical measures - an overlap *p*-value and an activation *Z*-score - are computed. The overlap *p*-value predicts likely upstream regulators based on significant overlap between dataset genes and known targets regulated by a transcription regulator or upstream regulator. The activation *Z*-score is used to infer likely activation states of upstream regulators based on comparison with a model that assigns random regulation directions. The purpose of the overlap *p*-value is to identify transcriptional regulators that are able to explain observed gene expression changes. The overlap *p*-value determines whether there is a statistically significant overlap between the dataset genes and the genes that are regulated by an upstream transcription regulator. It is calculated using Fisher’s Exact Test, and significance is generally attributed to *p*-values < 0.01. Since the regulation direction (“activating” or “inhibiting”) of a relationship is not taken into account for the computation of overlap *p*-values, the underlying network also includes findings without associated directional attribute, such as protein-DNA promoter binding. The primary purpose of the activation *Z*-score is to infer the activation states of predicted upstream transcription regulators. The basis for inference are the relationships in the molecular network that represent experimentally observed gene expression or transcription events, and are associated with a literature-derived regulation direction, which can be either “activating” or “inhibiting”. The statistical approach defining a quantity (*Z*-score) determines whether an upstream transcription regulator has significantly more “activated” predictions than “inhibited” predictions (*Z* > 0) or vice versa (*Z* < 0). The definition of upstream transcriptional regulator in this analysis is broad, namely any molecule that can affect the expression of other molecules, which means that upstream regulators can be almost any type of molecule, from transcription factors to microRNAs, kinases, compounds or drugs.

### Bacterial Cultivation

The reference strain *Campylobacter jejuni* wildtype 81-176 was pre-cultured on blood agar plates at 37°C in plastic jars with CampyGen gas packs from Oxoid (Thermo Scientific, Waltham, MA, United States) to generate a microaerobic atmosphere. After 48 h of incubation bacterial colonies were transferred into Mueller Hinton broth and incubated with shaking (200 rpm) at 37°C for 2 h under microaerobic conditions. Bacteria were centrifuged (5000 × *g*, for 2 min, at 10°C), resuspended in phosphate buffered saline (PBS; Gibco, Carlsbad, CA, United States) and adjusted to an optical density OD_600_ of one in the cell culture medium for the application of a defined multiplicity of infection (MOI) to the epithelial cell monolayers.

### Human Cell Culture Model

HT-29/B6-GR/MR cells (Bergann et al., 2009) were cultivated in 25 cm^2^ culture flask in RPMI 1640 culture medium (Sigma Aldrich, St. Louis, MO, United States) supplemented with 10% fetal calf serum (FCS, Gibco, Carlsbad, CA, United States), 1% penicillin/streptomycin (Corning, Wiesbaden, Germany), 300 μg/ml G418 BC (Invitrogen, Carlsbad, CA, USA), and 200 μL/ml hygromycin B (Biochrom GmbH, Berlin, Germany). Every seven days cells were passaged and then seeded on Millicell PCF filter membranes (Merck Millipore, Billerica, MA, United States) with a pore size of 3 μm, where they formed confluent epithelial monolayers. Six days after seeding the cells were washed twice with antibiotic-free cell culture medium supplemented with 10% heat-inactivated FCS. Seven days after seeding the cells reached transepithelial electrical resistances (TER values) of 600–900 Ω⋅cm^2^.The cells were treated with 100 µM trans-resveratrol (≥99% purity (HPLC), 3,4′,5-Trihydroxy-trans-stilben, 5-[(1E)-2-(4-Hydroxyphenyl)-ethenyl]-1,3-dihydroxybenzol, Sigma Aldrich, St. Louis, MO, United States) from the apical compartment 2 h before apical infection with *C. jejuni* (MOI 100).

### Electrophysiological Measurements of Barrier Function and Permeability Measurements

Transepithelial electrical resistance (TER) of the cell monolayers was measured under sterile conditions with chopstick electrodes (STX2, World Precision Instruments, Sarasota, FL, United States) and a volt-ohm meter (Clinical Physiology, Charité–Universitätsmedizin, Berlin). Measured TER values were corrected with the TER of an empty filter support and calculated on the surface area in square centimeters with the effective growth area of 0.6 cm^2^. In parallel, small molecule permeability was determined by measuring fluxes of the tracer fluorescein (332 Da, Sigma Aldrich, St. Louis, MO, United States) in 12-well plates. Fluorescent medium samples were taken from the basal compartment for 1 h (every 15 min), and the samples were subsequently analyzed by spectrophotometry (Tecan GmbH, Maennendorf, Switzerland). Permeability for fluorescein was calculated from the flux divided by the concentration difference.

### Cytokine-Stimulated *in vitro* Model

Epithelial cells were treated for 24 h with different concentrations and combinations of IFN-γ, TNF-α, and IL-1β (recombinant human cytokines, PeproTech, Rocky Hill, NJ, United States). Cytokines were added to the basolateral compartment. The next day, fluorescein (332 Da) flux measurements were performed in the 12-well plates for 1 h (every 15 min).

### *Campylobacter*-Infection and Resveratrol Treatment of the Colitis Mouse Model

Three-week-old IL-10^−/−^ mice with a C57BL/6j background were treated for eight weeks with an antibiotic cocktail containing ampicillin plus sulbactam (1 g/L; Dr Friedrich Eberth Arzneimittel, Ursensollen, Germany), ciprofloxacin (200 mg/L; Fresenius Kabi, Bad Homburg, Germany), imipenem (250 mg/L; Fresenius Kabi, Bad Homburg, Germany), metronidazole (1 g/L; Fresenius, Bad Homburg, Germany), and vancomycin (500 mg/L; Hikma Pharmaceuticals, London, United Kingdom) in autoclaved drinking water *ad libitum* to remove the commensal intestinal microbiota. Generation, housing, infection, and treatment of the IL-10^−/−^ mice were carried out under specific pathogen-free conditions in the animal facility of the Forschungseinrichtung für Experimentelle Medizin of the Charité–Universitätsmedizin Berlin. Two days before infection, treatment was started with trans-resveratrol (0.3 mg/ml [1.3 mM] resveratrol dissolved in 2% carboxy-methyl-cellulose) in drinking water provided *ad libitum*. Mice were infected on day 0 and day 1 by oral gavage with 10^9^ colony forming units (CFU) of *C. jejuni* (treatment group, named “resveratrol + *C. jejuni*”). The control group was *C. jejuni*-infected but received only drinking water containing the carrier-solution (placebo control, named “*C. jejuni*”). A small control group of naïve healthy mice was used for baseline measurements (named “control”). Mice were sacrificed at day 6 after infection and intestinal specimens were removed for fluorescein permeability or cytokine measurements. For cytokine assessment in organs, colon and mesenteric lymph nodes specimens were cultured in 24-well plates in serum-free RPMI 1640 culture medium supplemented with penicillin and streptomycin for 18 h at 37°C. IFN-γ and TNF-α concentrations were determined in the cell culture supernatant with the Mouse Inflammation Cytometric Bead Array (CBA; BD Biosciences, Heidelberg, Germany) using a FACSCanto II flow cytometer (BD Biosciences, Heidelberg, Germany).

### Ethical Statements

Animal experiments were carried out in accordance with the German Animal Protection Act and the ARRIVE guidelines. The study was approved by the ethics committee for animal welfare in Berlin, Landesamt für Gesundheit und Soziales (LAGeSo Berlin) under the LAGeSo approval number G0104/19.

### Western Blotting

Tight junction protein expression changes were analyzed by Western blotting. Proteins from cell culture experiments were lyzed using whole-cell lysis buffer, containing 10 mm Tris buffer (pH 7.5), 150 mm NaCl, 0.5% Triton X-100, 1% SDS, and one tablet Complete Protease Inhibitor Cocktail (Roche AG, Mannheim, Germany). After lysis, cells were scraped carefully from the filter supports, added to a reaction tube, incubated on ice for 30–60 min, and centrifuged for 30 min at 15000 x g and 4°C. After sonication and a further centrifugation step, protein quantification followed with the Pierce BCA kit (Thermo Scientific, Waltham, MA, United States). Electrophoresis was performed in 12.5% polyacrylamide gels and transferred to nitrocellulose membranes. After blocking for 2 h in 1% Polyvinylpyrrolidone (PVP40; Sigma Aldrich, St. Louis, MO, United States) and 0.05% Polysorbate 20 (Tween20; Thermo Scientific, Waltham, MA, United States) primary antibodies raised against human occludin, claudin-2 (1:1000; Sigma Aldrich, St. Louis, MO, United States), claudin-1, −4, −5, −7, −8 (1:1000; Invitrogen, Carlsbad, CA, United States), and *β*-actin (1:10000; Sigma Aldrich, St. Louis, MO, United States) as loading control, were incubated slewing at 4°C overnight. Next day, membranes were washed with TBST buffer and incubated with secondary peroxidase-conjugated antibodies goat anti-rabbit IgG or goat anti-mouse IgG (Jackson ImmunoResearch, Ely, United Kingdom) in TBST supplemented with 1% milk powder (Sigma Aldrich, St. Louis, MO, United States) for 2 h at room temperature. For protein detection, membranes were incubated for 2–5 min in SuperSignal West Pico PLUS Stable Peroxide Solution (Thermo Scientific, Waltham, MA, United States) and visualized using a Fusion FX7 imaging system (Vilber Lourmat Deutschland GmbH, Eberhardzell, Germany). Image Studio Lite version 5.2 was used for densitometric analysis.

### Immunofluorescence Microscopy of Tight Junction Proteins and Apoptosis

Cells grown on filter supports were rinsed twice with PBS and fixed with 2% paraformaldehyde (Electron Microscopy Sciences, Hatfield, PA, United States) for 30 min at room temperature. After permeabilization for 7 min with 0.5% Triton X-100 (Sigma Aldrich, St. Louis, MO, United States), cells were blocked 10 min at room temperature with blocking solution containing 5% goat serum (Gibson, Carlsbad, CA, United States), 0.05% Triton X-100, and 1% bovine serum albumin (BSA; Sigma Aldrich, St. Louis, MO, United States). Afterwards, cells were incubated for 1 h at 37°C with primary antibodies anti-occludin (1:100; Sigma Aldrich, St. Louis, MO, United States), anti-claudin-5 (1:100; Invitrogen, Carlsbad, CA, United States), and anti-zonula occludens protein-1 (ZO-1, 1:100; BD Biosciences, Franklin Lakes, NJ, United States) followed by the secondary anti-rabbit or anti-mouse antibody conjugated to Alexa-Fluor 488 or 594 (1:500; Invitrogen, Carlsbad, CA, United States) for 1 h at 37°C. For apoptosis detection, cells were stained with the TUNEL kit (*In situ* Cell Death Detection Kit, Roche AG, Mannheim, Germany) according to manufacturer’s instructions. Nuclei were stained with 4,6-diamidino-2-phenylindole (DAPI; 1:1000; Roche AG, Basel, Switzerland). Visualization was performed by confocal laser-scanning microscopy (CLSM; Zeiss LSM780, Jena, Germany).

### Statistics

All data are expressed as mean values ± standard error of the mean (SEM). Statistical analysis was performed using GraphPad Prism version 8.0. For comparison of two groups, Student’s *t*-test and for comparison of more than two groups one-way ANOVA with Bonferroni adjustment for multiple comparisons was used. Data that were not normally distributed were analyzed using the Mann Whitney *U*-test. A value *p <* 0.05 was considered to be statistically significant.

## Results

### Bioinformatics Prediction for the Suitability of Resveratrol

In a bioinformatics calculation from our existing dataset of a differential RNA expression profile in *C. jejuni*-infected human colonic mucosa, we searched putative suitable compounds for the treatment of campylobacteriosis. In the Ingenuity Pathway Analysis, resveratrol was found to show an inhibited pathway profile in the Upstream regulator analysis with 1.09 E^-5^ *p*-value of overlap and an activation *Z*-score of −2.8 (negative score, pointing to inhibited pathways/downstream targets), indicating that the presence of resveratrol could counter-regulate the *C. jejuni*-induced changes. As an Upstream regulator, resveratrol shows comparable values for inhibited pathways like the nutraceutical polyphenol curcumin with a *p*-value of 2.06 E^-5^ or quercetin with a *p*-value 1.95 E^-5^. By contrast, activated pathways with positive Z-scores and comparable *p*-values were calculated for pro-inflammatory cytokines, e.g. IL-17; overlap *p*-value 1.71 E^-5^, *Z*-score +3.7 (with activated pathways/downstream targets). The cytokines with the highest significance for activated pathways IFN-γ and TNF-α showed activation *Z*-scores of +9.6 and +9.0 respectively, and both were considered as the major cytokine pathways in campylobacteriosis, as reported previously (Bücker et al., 2018). Sequencing data are deposited in NCBI’s Gene Expression Omnibus; GEO ID GSE88710.

### Epithelial Barrier Dysfunction After *Campylobacter jejuni* Infection in HT-29/B6 Monolayers Was Rescued by Resveratrol

**FIGURE 1:**
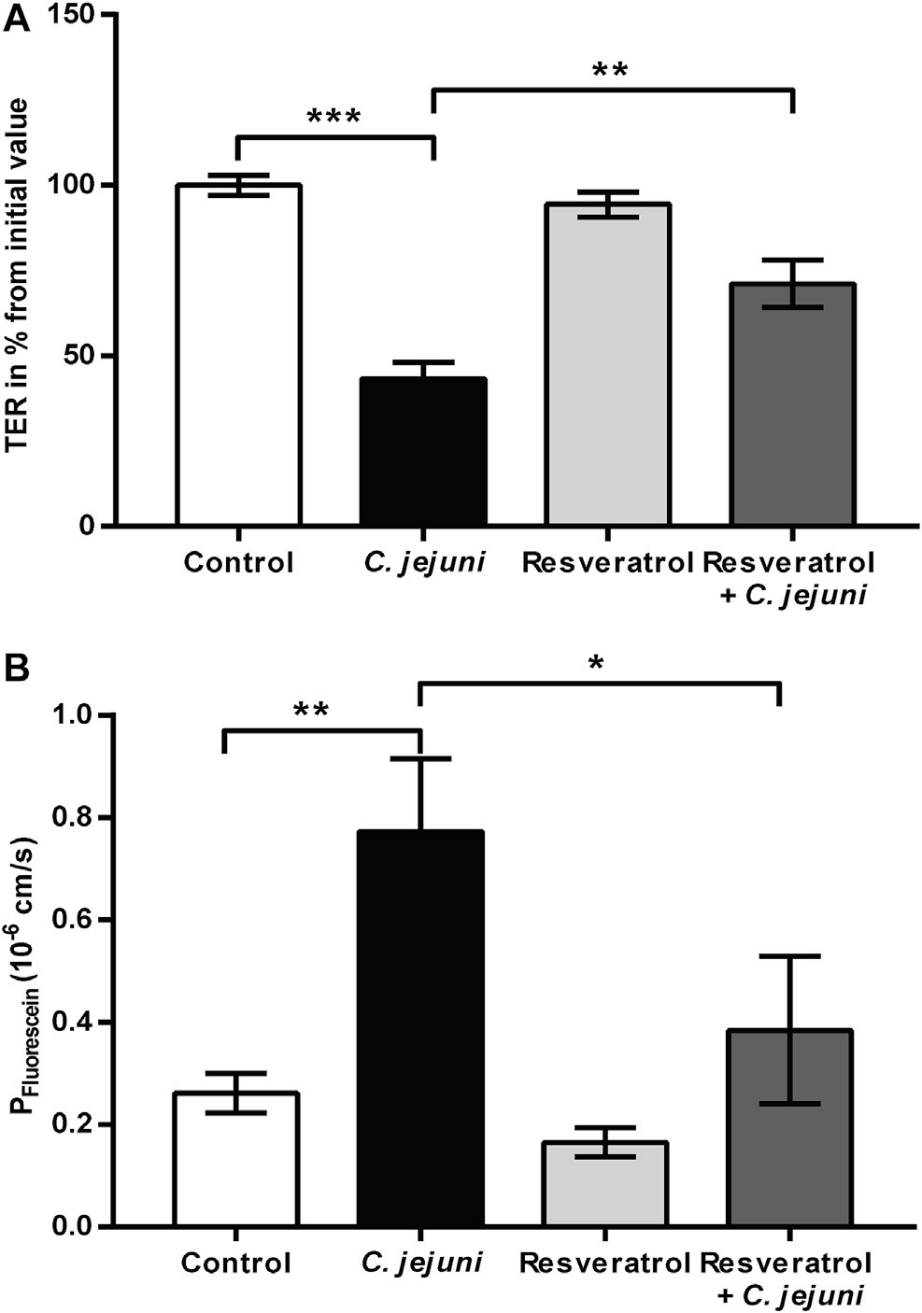
Epithelial barrier function and macromolecule flux after *Campylobacter jejuni* infection in epithelial cell monolayers and after treatment with resveratrol. Human intestinal epithelial HT-29/B6 cells were treated with 100 µM of resveratrol from apical and infected apically with *C. jejuni* (multiplicity of infection; MOI 100). Transepithelial electrical resistance (TER) was measured with chopstick electrodes 48 h after the infection **(A)** Resveratrol inhibits the *C. jejuni*-induced decrease in TER *in vitro*. Control values after 48 h were set at 100% (n = 9–10, **p < 0.01, one-way ANOVA with Bonferroni correction). **(B)** Resveratrol prevents *C. jejuni*-induced increase in permeability to fluorescein (332 Da) *in vitro* (*n* = 10, *p < 0.05, **p < 0.01, one-way ANOVA with Bonferroni correction).

The induction of epithelial barrier impairment by *C. jejuni* has already been described (Bücker et al., 2018). The inhibitory effect of resveratrol on the *C. jejuni*-induced barrier dysfunction was tested here for the first time *in vitro*. In our infection experiments with *C. jejuni*, pharmacological relevant concentrations of resveratrol were chosen to investigate the actions of resveratrol on the intestinal epithelium without directly inhibiting the bacteria. Administration of 100 µM resveratrol (= 23 μg/ml) alone to HT-29/B6 monolayers did not induce any changes in TER over two days of incubation (Figure 1A). Infection of HT-29/B6 monolayers with *C. jejuni* from the apical side revealed a drop in TER to 43 ± 5% of the initial value after 48 h (Figure 1A). This barrier-breaking effect was prevented by the addition of resveratrol (Figure 1A).

### Paracellular Flux Marker Fluorescein Showed Increased Permeability After *Campylobacter jejuni* Infection and its Reversal by Resveratrol Treatment

The TER represents the permeability for ions, whereas the paracellular leakiness or tightness of an epithelium towards larger molecules is represented by marker fluxes. In order to characterize the barrier defect by *C. jejuni* on the paracellular pathway, we used the flux marker fluorescein (332 Da). Flux measurements between the apical and basolateral side of HT-29/B6 monolayers revealed an increase in permeability for fluorescein in *C. jejuni*-infected monolayers, whereas the resveratrol-treated and infected monolayers showed reduced fluorescein permeability in comparison to the *C. jejuni*-infected group. The resveratrol-treated *C. jejuni*-infected cells showed comparable results to the uninfected untreated controls (*p* > 0.05) (Figure 1B). Thus, the TER and fluorescein measurements indicate that resveratrol prevented the disturbance in epithelial barrier function induced by *C. jejuni*.

### Tight Junction Expression and its Subcellular Distribution Change During *Campylobacter jejuni* Infection and Resveratrol Treatment in HT-29/B6 Monolayers

**FIGURE 2:**
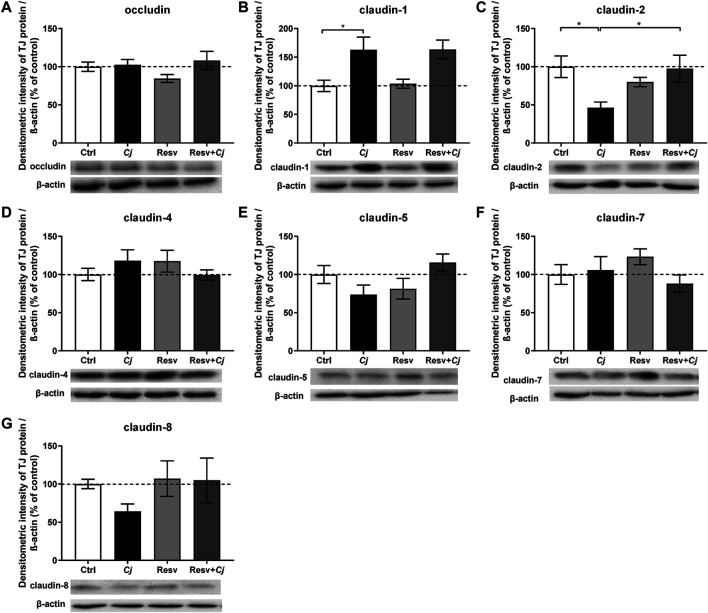
Expression of tight junction proteins after *Campylobacter jejuni* infection and resveratrol treatment. Epithelial tight junction protein expression changes under *C. jejuni* (*Cj*) infection and resveratrol (Resv) treatment *in vitro*. Proteins from HT-29/B6-GR/MR cell monolayers were isolated after 48 h *C. jejuni* infection with or without 100 µM resveratrol treatment for protein expression analysis by Western blotting **(A)** occludin **(B)** claudin-1 **(C)** claudin-2 **(D)** claudin-4 **(E)** claudin-5 **(F)** claudin-7, and **(G)** claudin-8 expression were quantified by densitometric analysis of Western blots, normalized to *β*-actin. Untreated uninfected cells were set as controls (Ctrl) at 100% (*n* = 5–10, *p < 0.05, one-way ANOVA with Bonferroni correction).

The molecular basis of an impaired leak pathway is often based on the expression and/or distribution of TJ proteins. To detect changes in TJ protein expression in HT-29/B6 cell monolayers, we used Western blotting with antibodies raised against occludin and claudins (Figure 2). Expression of occludin (Figure 2A), claudin-4 (Figure 2D) and claudin-7 (Figure 2F) in HT-29/B6 monolayers was unaffected by *C. jejuni* infection or resveratrol treatment. Although the expression level of claudin-5 (Figure 2E) and claudin-8 (Figure 2G) showed a tendency towards a reduction after infection and to be reversed by resveratrol treatment, this did not reach statistical significance. Moreover, infection did not appear to induce the cleavage of occludin. However, claudin-2 protein expression was decreased and fully restored to control levels by resveratrol (Figure 2C), while claudin-1 expression was increased after infection and was unchanged by resveratrol (Figure 2B).

**FIGURE 3:**
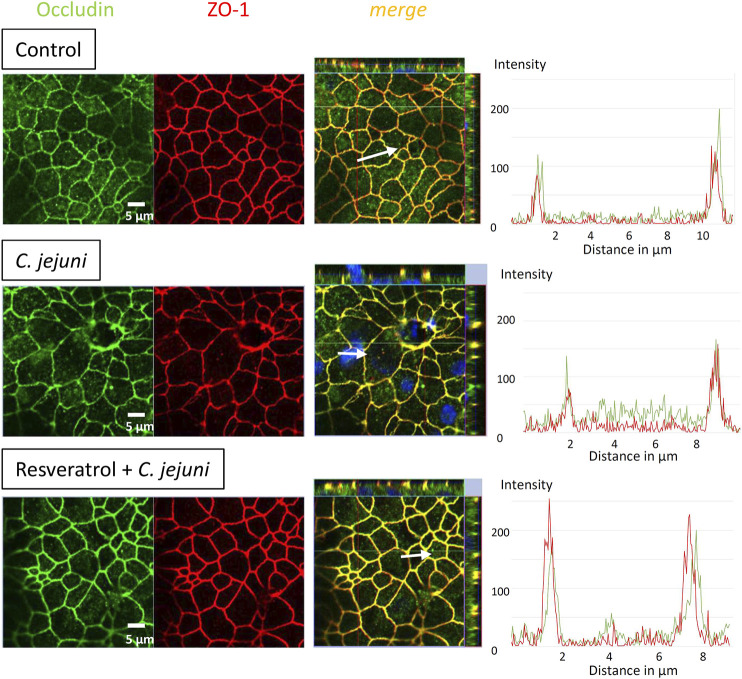
Subcellular tight junction protein distribution of occludin under *Campylobacter jejuni* infection and resveratrol treatment. Resveratrol mitigates *C. jejuni*-induced re-distribution of occludin. HT-29/B6-GR/MR cells were treated with resveratrol and infected with *C. jejuni* from apical. 48 h post-infection monolayers grown on filter inserts were stained with antibodies against occludin (green) and zonula occludens protein-1 (ZO-1; red). Nuclei were stained with 4,6-diamidino-2-phenylindole (DAPI) in blue (bar 5 µm). Intensity plots point to signals of the TJ proteins indicated with white arrows in the merge pictures. The intensity signals appear highest in the TJ domain with a proper co-localization under control conditions and vary under infection and treatment conditions along the white arrows over single cells.

**FIGURE 4:**
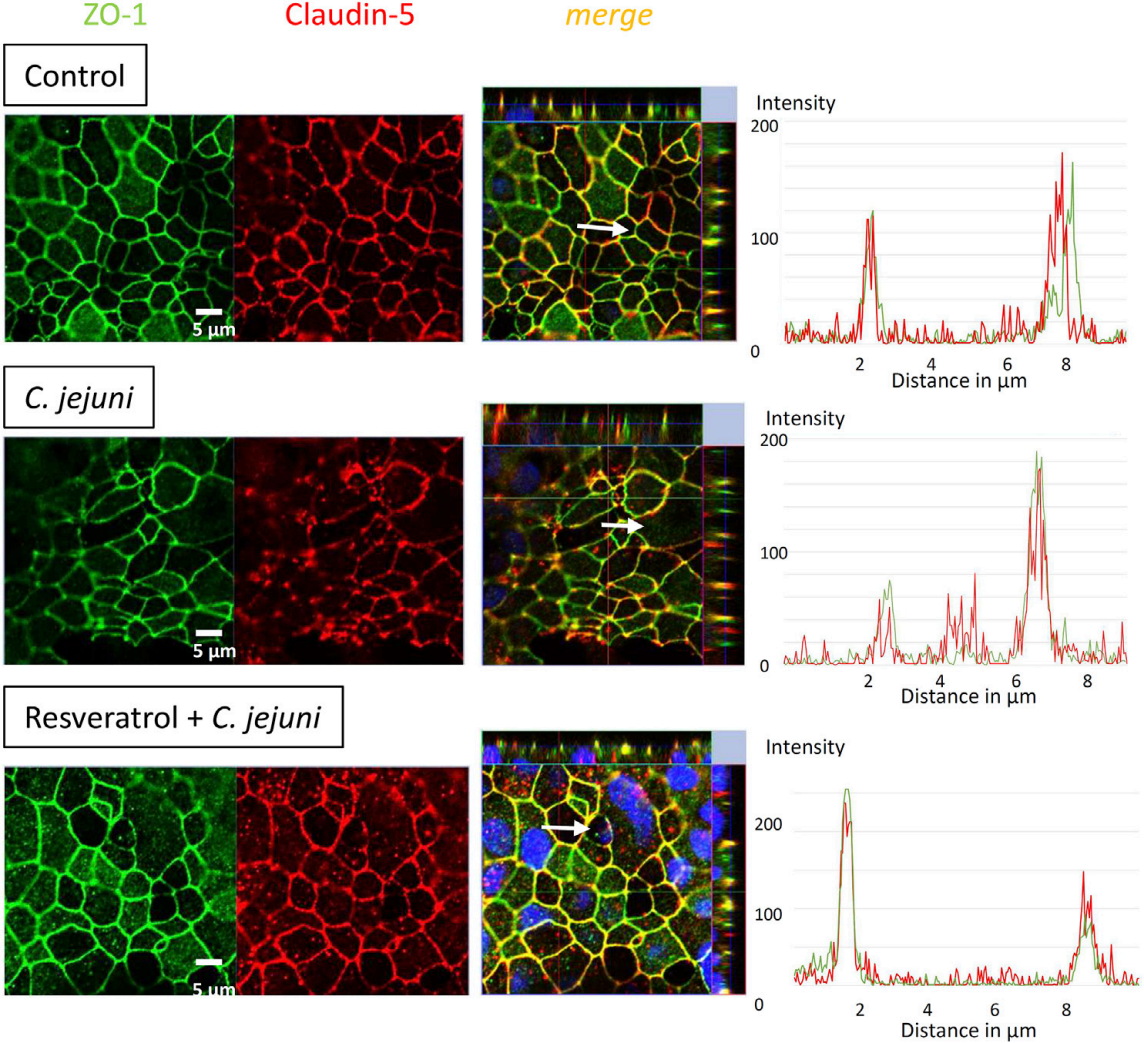
Subcellular tight junction protein distribution of claudin-5 under *Campylobacter jejuni* infection and resveratrol treatment. Resveratrol mitigates *C. jejuni*-induced re-distribution of claudin-5. HT-29/B6-GR/MR cells were treated with resveratrol and infected with *C. jejuni* from apical. 48 h post-infection monolayers were stained with antibodies against claudin-5 (red) and ZO-1 (green). Nuclei were stained blue with DAPI (bar 5 µm). Intensity plots point to signals of the TJ proteins along the white arrows in the merge pictures. The intensity signals appear highest in the TJ domain with a proper co-localization under control conditions and vary under infection and treatment conditions with the white arrows depicted over single cells.

To determine the effects of *C. jejuni* and resveratrol on the localization of TJ proteins, we performed fluorescent immunostaining for confocal laser-scanning microscopy (CLSM) and analyzed the micrographs using the intensity plot function of the CLSM software. After infection with *C. jejuni* the TJ proteins, occludin and claudin-5 were redistributed off the TJ domain of the epithelial cells (Figures 3, 4). Occludin signals were found to be intracellular and no longer co-localized with ZO-1 in the TJ strands (Figure 3). Even more pronounced was the redistribution of claudin−5 off the TJ domain and the appearance of intracellular claudin−5 signals (Figure 4). In Figures 3, 4, intensity plots point to increased intracellular signals of occludin or claudin−5 in response to infection, which were diminished after resveratrol treatment. The change in the expression profiles of claudin−1 and −2 (and −5 and −8 by trend), and the subcellular signals of occludin and claudin-5 detected by CLSM, resembles a restitution reaction of the epithelium as described previously during *C. jejuni* infection and also observed during the induction of apoptosis (Bojarski et al., 2004; Amasheh et al., 2010; Rosenthal et al., 2017; Bücker et al., 2018; Butkevych et al., 2020).

### Induction of Epithelial Apoptosis by *Campylobacter jejuni* and its Recovery by Resveratrol

**FIGURE 5:**
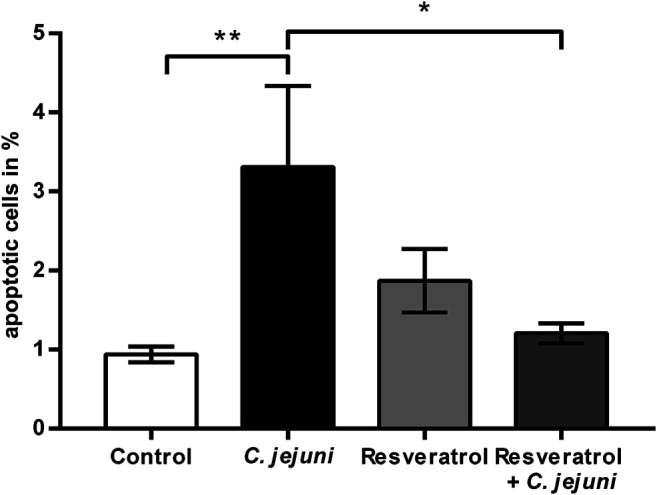
Apoptosis induction as barrier-relevant event and protection by resveratrol. *Campylobacter jejuni*-induced apoptosis was inhibited by resveratrol. HT-29/B6-GR/MR cells treated with 100 µM resveratrol were infected from the apical side with *C. jejuni*. 48 h post-infection cells were stained with TUNEL reagent to visualize apoptotic cells. 4,6-diamidino-2-phenylindole (DAPI) was used for staining of the nuclei. Apoptotic cells were counted in every filter in six different low power fields (approximately 500 cells) to calculate the apoptotic ratio (n = 6, *p < 0.05, **p < 0.01, one-way ANOVA with Bonferroni correction).

In addition to TJ disruption during *C. jejuni* infection, the induction of epithelial apoptosis represents a pathomechanism which is relevant to the disruption of barrier function. *C. jejuni* induces epithelial apoptosis via cleavage of caspase-3 (Bücker et al., 2018; Lobo de Sá et al., 2019; Mousavi et al., 2019; Butkevych et al., 2020). We therefore studied the possible therapeutic benefit of resveratrol on the induction of apoptosis. TUNEL staining was applied and visualized using CLSM. *C. jejuni* infection increased the apoptotic ratio in the HT-29/B6 cell monolayers 3-fold in comparison to controls. Resveratrol treatment of the *C. jejuni* infected monolayers reduced the apoptotic ratio to the level in controls (*p* > 0.05; Figure 5). Thus, the ability of resveratrol to prevent epithelial cell death as well as restore TJ protein localization explains its efficacy in preventing the epithelial barrier defects induced by *C. jejuni* infection.

### *In vitro* Effects of Pro-inflammatory Cytokines and Their Prevention by Resveratrol

**FIGURE 6:**
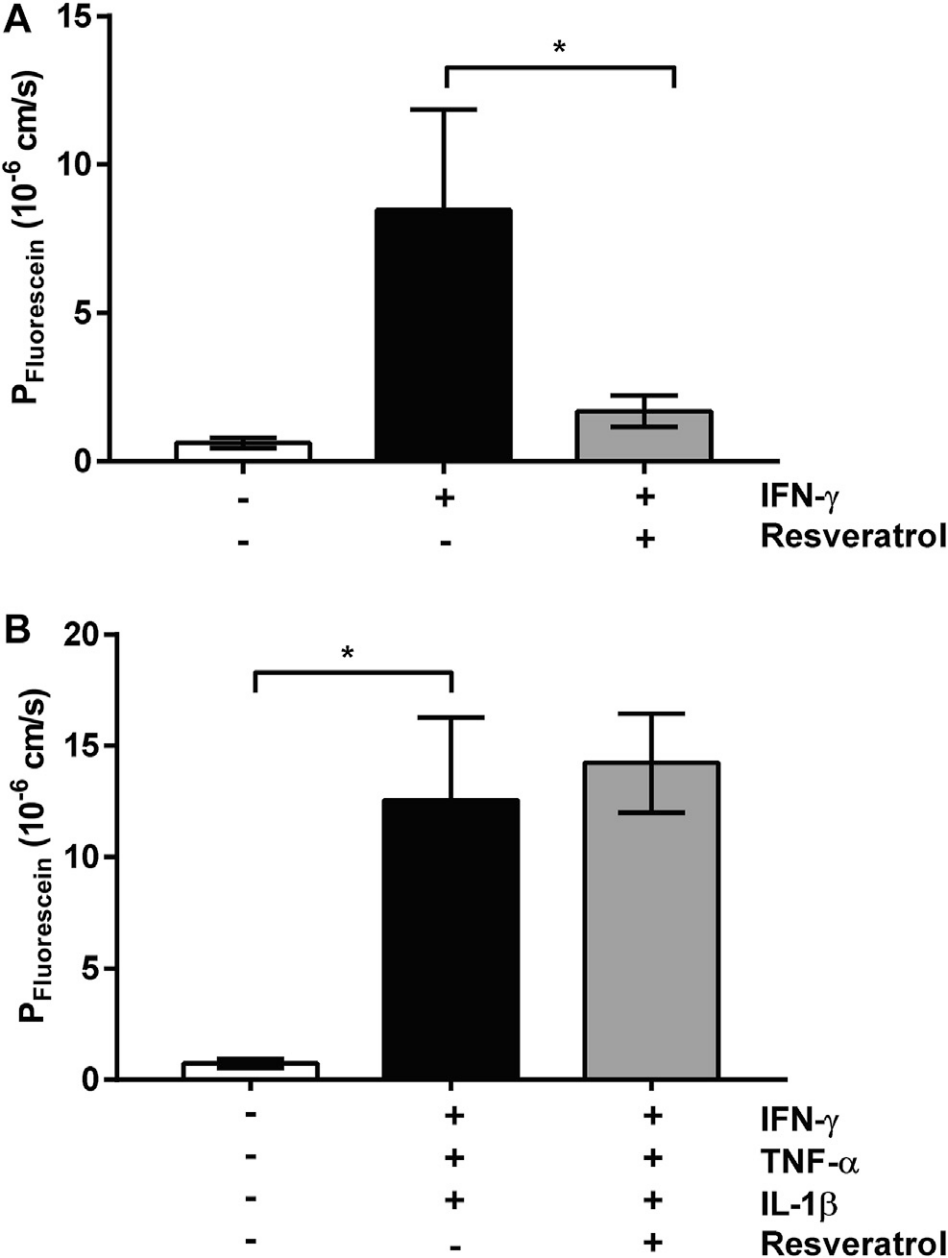
*In vitro* stimulation model with cytokines and resveratrol. Cytokine-induced barrier dysfunction from the subepithelial compartment of HT-29/B6-GR/MR cell monolayers. Resveratrol treatment was performed from the apical side 2 h before cytokine addition. Fluorescein permeability displays barrier-breaking effects for **(A)** 50 ng/ml IFN-γ and **(B)** high-dose cytokine cocktail containing 100 U/mL TNF-α, and 100 ng/ml IL-1β, 50 ng/ml IFN-γ (*n* = 3–8, *p < 0.05, ***p < 0.001, one-way ANOVA with Bonferroni correction).

We analyzed the effect of resveratrol on cytokine-induced barrier disruption using a cell model of the *leaky gut*. We mimicked subepithelial cytokine release in the epithelial cell model by adding the major cytokines found in campylobacteriosis, namely of IFN-γ, TNF-α and IL-1β. This model resembles the *leaky gut* situation, where stimulated subepithelial immune cells release these cytokines. When the single cytokine 50 mg/ml IFN-γ was added, fluorescein permeability increased and this effect was attenuated by resveratrol (Figure 6A). By contrast, addition of IFN-γ, TNF-α and IL-1β at high doses induced an increase in paracellular permeability for fluorescein, but no recovery was obtained with 100 µM resveratrol (Figure 6B). These experiments indicate that the effects of cytokines at submaximal but not at maximal concentrations are counteracted by resveratrol.

### *In vivo* Confirmation of Barrier Impairment of the Paracellular Pathway in the *C. jejuni*-Infected Mouse Model and its Prevention by Resveratrol

**FIGURE 7:**
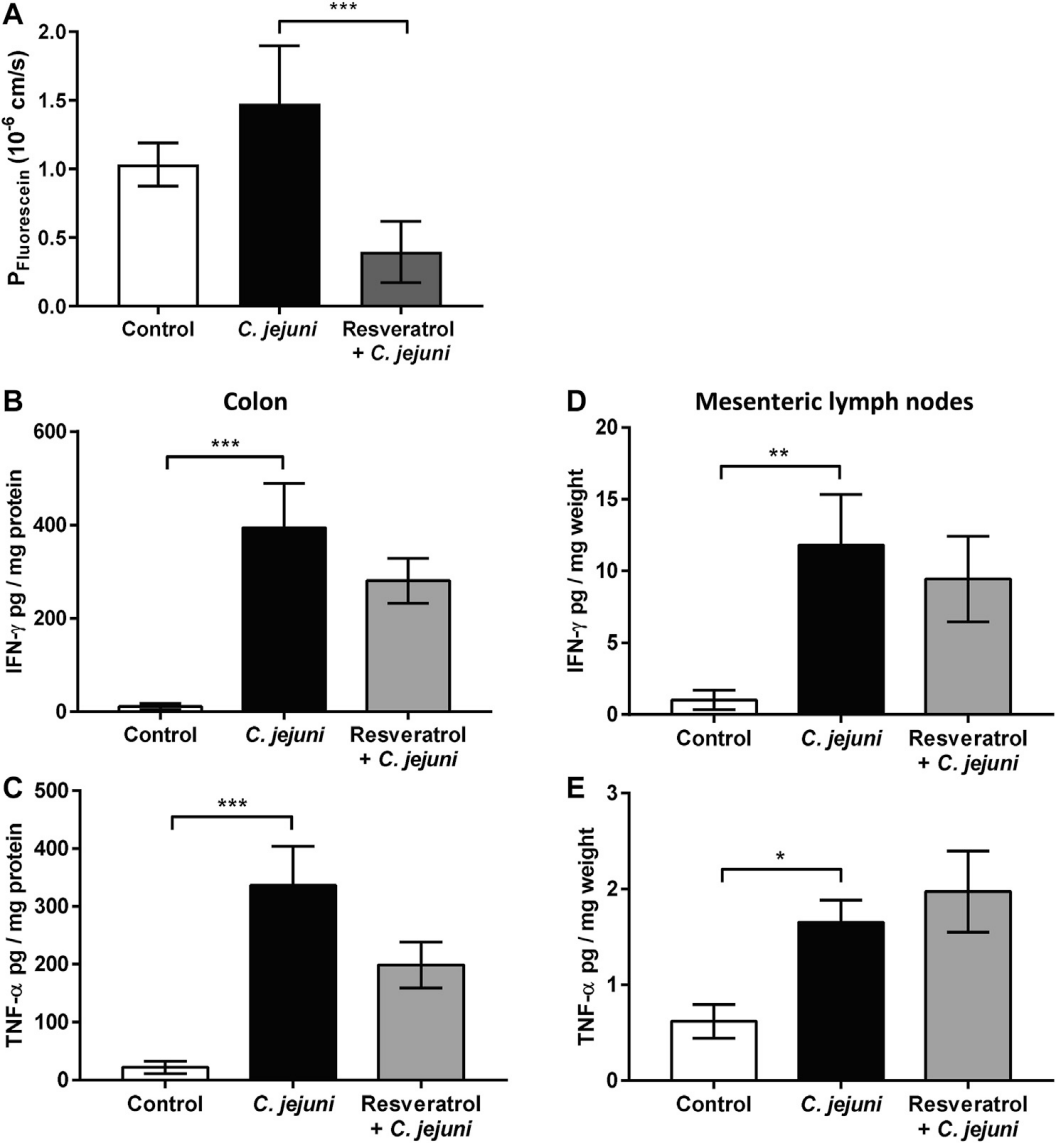
*In vivo* mouse infection model and oral treatment with resveratrol. Barrier function and simulated antigen influx by fluorescein in the *Campylobacter jejuni*-infected mouse colon. Secondary abiotic IL-10^−/−^ mice were infected with *C. jejuni*, treated with resveratrol via the drinking water and sacrificed at day 6 post-infection **(A)** Colon specimens of *C. jejuni*-infected and resveratrol treated mice were mounted into Ussing chambers and flux measurements of fluorescein were performed in mucosal to serosal direction **(B and C)** release of pro-inflammatory cytokines of mouse organs from colon and **(D and E)** mesenteric lymph nodes. Organ specimens were cultured in cell culture medium and supernatant was analyzed by Cytometric Bead Array for **(B and D)** IFN-γ and **(C and E)** TNF-α concentrations (*n* = 4–17, *p < 0.05, **p < 0.01, ***p < 0.001, Mann Whitney *U*-test).

To study the effectiveness and the mechanism of the effect of resveratrol on barrier function seen *in vitro* and *in vivo*, the *C. jejuni*-infected secondary abiotic IL-10^−/−^ mouse model was used. Estimation of the intestinal epithelial leakiness was evaluated, by measuring fluxes of the paracellular flux marker fluorescein across colon from *Campylobacter*-infected mice mounted in Ussing chambers. Paracellular permeability for fluorescein was higher in colon mucosae of *C. jejuni*-infected mice compared with resveratrol-treated mice at day 6 post-infection (*p* < 0.05; Figure 7A), indicating that resveratrol treatment rescues colonic epithelial barrier function following *C. jejuni* infection.

Since mucosal cytokine release could be counter-regulated by resveratrol, we also assessed cytokine production during infection, in our mouse model. The concentrations of IFN-γ and TNF-α were elevated in colon mucosae at day 6 post-infection, but did not differ between the *C. jejuni*-infected placebo and the resveratrol-treated groups (Figures 7B,C). The same was true of cytokine levels in the mesenteric lymph nodes (Figures 7D,E). Thus, in keeping with our *in vitro* results, the anti-*Campylobacter* effect of resveratrol seems to be based more on improving barrier function at the epithelial level than by attenuating cytokine release.

To comply with the guidelines of the 3R principles to reduce the number of animal experiments, we used animal biopsies and experimental *in vivo* data from an ongoing study in collaboration (Heimesaat et al., 2020).

## Discussion

### Resveratrol - A Valuable Compound in *Campylobacter jejuni* Infection

Based on the functional effects of resveratrol reported in the literature as well as on our bioinformatics prediction, this natural compound was deemed to be effective in antagonizing the *C. jejuni*-induced changes in host epithelial cells. Direct antimicrobial effects on different pathogenic bacteria, including *Campylobacter,* have been reported at higher concentrations of resveratrol, but not at lower concentrations (minimum inhibitory concentration (MIC) of 300 μg/ml) (Klančnik et al., 2017). In our *in vitro* assays, the concentration of resveratrol required to block *C. jejuni*-induced barrier defects was 100 µM (23 μg/ml), which was lower than the MIC required to act directly against *C. jejuni*. Likewise, the resveratrol concentration used for the *in vivo* experiments was close to, but lower than, the MIC of *C. jejuni* 81-176 with 456.5 μg/ml (Heimesaat et al., 2020). Thus, the protective effects of resveratrol appear to be targeted to the host response rather than to a direct anti-bacterial effect in the infected animals (Heimesaat et al., 2020).

In *C. jejuni*-infected patients, profound intestinal epithelial barrier dysfunction together with inflammation of the colonic mucosa are prominent features of this diarrheal disease, which is characterized by watery or bloody diarrhea, often accompanied by fever and elevated CRP values (Nielsen et al., 2012; Bücker et al., 2018). The main diarrheal mechanism in *C. jejuni* infection is based on the *leak flux* type of diarrhea, in which water and solutes traverse the leaky epithelium towards the intestinal lumen (Bücker et al., 2018). Concomitant with this change in fluid and electrolyte transport, the *leaky gut* facilitates antigen influx via the dysfunctional epithelial barrier, which results in an accelerated immune response and further disruption of barrier function (a self-sustaining feedback loop).

Underlying our hypothesis and planned approach for treating and preventing campylobacteriosis and its sequelae, such as irritable bowel syndrome (IBS), reactive arthritis and the Guillain-Barré syndrome, is the idea that a compound might strengthen the epithelial barrier, thereby preventing intestinal leakiness and antigen entry from the lumen. In our *in vitro* experiments, resveratrol restored epithelial barrier function following *C. jejuni* infection and is therefore a promising nutraceutical. This suitability was predicted in the bioinformatics RNA-Seq data from campylobacteriosis patients by Ingenuity Pathway Analysis software. The Ingenuity Pathway Analysis prediction tool for activated or inhibited pathways and potentially active compounds (upstream analysis) in different diseases compares differential expressed genes in our *Campylobacter* dataset with a database of numerous available expression datasets from the literature. The information from the literature enables predictions not only for the pathogenesis and the significant or associated signaling pathways, but also on possible compounds that were already found to be effective in other diseases with similar patterns of RNA expression changes to that in campylobacteriosis. Although ancient knowledge from traditional healing arts points to the usefulness of plants containing resveratrol, questions about pharmacokinetics, bioavailability and enteral stability, as well as the adverse effects of resveratrol preparations, need to be addressed by clinical trials.

### Apoptosis Induction and Tight Junction Protein Changes in *Campylobacter* Infection and Resveratrol Treatment

The anti-apoptotic effect of resveratrol is of high importance. The *C. jejuni* infection not only affects the TJ disruption-dependent leak pathway, but also the unrestricted pathway by barrier-relevant cytotoxicity. In our cell monolayer infection experiments, the ratio of apoptotic events was increased 3-fold and could be reduced to control values in the resveratrol treated group. An increase in apoptosis to this extent in an epithelial layer is barrier-relevant (Bojarski et al., 2004; Butkevych et al., 2020) and can explain, at least in part, the functional barrier defects as quantified by our measurements of TER and fluorescein permeability. Anti-apoptotic effects of resveratrol have been reported in H_2_O_2_-stimulated Caco-2 cells (Wang et al., 2016), and were confirmed in the present study. *Campylobacter jejuni*-infected IL-10^−/−^ mice treated with resveratrol showed lower apoptotic cell numbers in the colon when compared with placebo-treated *C. jejuni*-infected mice (Heimesaat et al., 2020). Nevertheless, several studies and clinical trials showed that resveratrol also induced apoptosis in cancer cells (Berman et al., 2017; Singh et al., 2017; El-Kott et al., 2019). Besides the induction of apoptosis induction, the other main component of the functional barrier impairment can be explained by changes in TJ protein expression and subcellular redistribution of TJ proteins (Chen et al., 2006; Bücker et al., 2018). After infection with *C. jejuni,* HT-29/B6 cell monolayers showed a redistribution of barrier-maintaining occludin and claudin-5 off the TJ domain into intracellular compartments of the epithelial cells. A similar pattern of redistribution of occludin and claudin-5 has been reported in other *in vitro* infection experiments with *C. jejuni* (Chen et al., 2006; Rees et al., 2008; Lobo de Sá et al., 2019) and in colon tissues from campylobacteriosis patients (Bücker et al., 2018; Harrer et al., 2019). This redistribution will contribute to epithelial barrier dysfunction via the paracellular leak pathway, and provides the structural basis of the mechanism underlying *leak flux* diarrhea (Bücker et al., 2018).

A discrepancy was seen in our Western blot studies of the downregulation of channel-forming claudin-2 and the upregulation of barrier-forming claudin-1. The claudin-1 paradox has previously been described in *C. fetus* and *C. jejuni* infection, in which claudin-1 showed increased expression (while TER was decreased). The explanation for this paradox was that claudin-1 does not functionally assemble into the epithelial TJ strands but remains in intracellular compartments (Bücker et al., 2017; Bücker et al., 2018; Lobo de Sá et al., 2019; Butkevych et al., 2020). This can be interpreted as the start of a counter-reaction or recovery reaction 1–2 days post infection after the primary TJ dysregulation in the first phase of infection. By contrast, claudin-2 downregulation is assumed to be functionally masked by the other structural alterations.

### *Campylobacter jejuni* Infection in Stimulated Cell Cultures *in vitro* and Campylobacteriosis in Mice

Pro-inflammatory cytokines such as TNF-α (Amasheh et al., 2010; Luettig et al., 2016) trigger dysregulation of TJ proteins as well as inducing apoptosis, and the *mucosal cytokine storm* is prominent in the pathogenesis of campylobacteriosis (Al-Salloom et al., 2003; Edwards et al., 2010; Bücker et al., 2018). We therefore investigated the role of cytokines in causing barrier dysfunction during *C. jejuni* infection in *leaky gut* models.

*Leaky gut* models to study *Campylobacter* infection have previously employed monolayers of human colon cell lines co-cultured with M1-macrophages derived from human PBMCs (Bücker et al., 2018), or epithelial monolayers co-cultured with the stimulated macrophage-like cell line THP-1 (Lobo de Sá et al., 2019; Butkevych et al., 2020). Cell culture filter supports or transwell systems allow application of bacteria, cytokines, compounds or inhibitors from the apical or basolateral surfaces. For example, when investigating the immune-modulating effect of curcumin on the *C. jejuni* infection, basolateral located THP-1 cells were infected directly, without infecting the epithelial cell monolayers, simulating cytokine-induced impairment of the barrier function (Lobo de Sá et al., 2019). In this model, the addition of curcumin abolished barrier dysfunction in an NFκB-dependent manner (Lobo de Sá et al., 2019). The THP-1 model could not be applied in the present study as in this blood cancer cell line, resveratrol causes cell cycle arrest and induces apoptosis (Feng et al., 2019). For this reason, an alternative model with the addition of cytokines was used. Since Rees and colleagues showed that 100 U/mL IFN-γ accelerates barrier impairment synergistically with *C. jejuni* in infected Caco-2 cells (Rees et al., 2008), and our Ingenuity Pathway analysis indicated the importance of the IFN-γ pathway, we stimulated HT-29/B6 monolayers with IFN-γ (50 ng/ml). We found that resveratrol antagonized the IFN-γ-pathway resulting in an improvement of barrier function. In further experiments, barrier defects after maximal stimulation with a cytokine cocktail of TNF-α, IL-1β and IFN-γ could not be antagonized by resveratrol. This suggests that the pharmacological efficacy of resveratrol is primarily barrier-improving rather than immunomodulatory.

In addition to co-culture infection and direct cytokine exposure, the use of animal models is the standard method to gain information about the entire immune system after intestinal barrier breakdown during enteropathogenic infection (Gölz et al., 2015; von Klitzing et al., 2017; Heimesaat et al., 2019). In our present mouse model of campylobacteriosis, infected secondary abiotic IL-10^−/−^ mice suffered from acute enterocolitis within one week after infection (Haag et al., 2012), as indicated by bloody diarrhea and wasting. The underlying massive immune overreaction is based on the subepithelial cytokine storm induced by the LOS of the invasive campylobacters, suggesting that the pathogenicity of *C. jejuni* is predominantly based on LOS translocation into the subepithelium, whereby the influx of further LOS from the lumen in the *leaky gut* situation may amplifies mucosal inflammation. Given that the LOS of *C. jejuni* targets the toll-like receptor 4 (TLR4), the major role of LOS in the induction and progress of campylobacteriosis was supported by the fact that *C. jejuni* infection induced significantly milder enterocolitis symptoms in TLR4-deficient mice. This was confirmed independently by different research groups in their individual murine models of camyplobacteriosis (Otto et al., 2012; Haag et al., 2012; Stahl et al., 2014; Stahl & Vallance, 2015; reviewed by Mousavi et al., 2020). Most importantly, a general pharmacological mechanism of action against the inflammation induced by *C. jejuni* includes the blockage or antagonism of TLR4 signaling pathways, which should counter-regulate the pathological consequences of the leaky gut. This kind of immune induction via a TLR4-dependent pathway might be inhibited by resveratrol in our mouse experiments. In support of this notion, *C. jejuni-*induced inflammatory responses were significantly decreased by the polyphenol curcumin (Lobo de Sá et al., 2019), which is a potent TLR4 antagonist and inhibits LOS-mediated immune responses (Zhao et al., 2011).

However, the direct impact of resveratrol on cytokine pathways appears to be effective only in the case of moderate barrier dysfunction, such as that induced by IFN-γ treatment. Similar results were obtained in lymphocytes, in which cytokine production increased rather than decreased after resveratrol treatment (Gao et al., 2001). By contrast, production of IL-6 was downregulated in macrophages after resveratrol treatment (Zhong et al., 1999) whereas in mice with Dextran Sodium Sulfate (DSS)-induced colitis resveratrol decreased IL-6 release but increased TNF-α release (Mayangsari and Suzuki, 2018). Nevertheless, resveratrol restored intestinal barrier function in mice with DSS-induced colitis (Mayangsari and Suzuki, 2018). One interpretation of our results is that resveratrol does not function as an immunosuppressive agent, but rather as an immune-modulating compound acting on weakly affected cytokine pathways such as that dependent on IL-6. Therefore, the *C. jejuni* LOS-induced immune activation of TLR4, as occurs in our mouse model, might be modulated or in part inhibited by resveratrol, thereby contributing to the restoration of barrier dysfunction and avoidance of the *leaky gut*.

### Strengthening the Epithelial Barrier for Prevention of the Leaky gut

Resveratrol has not only immune-modulatory functions, but also can reduce oxidative stress and influences several pathways in intestinal homeostasis (Singh et al., 2010; Yao et al., 2010; Yao et al., 2015; Cao et al., 2019). In our epithelial cell monocultures infected with *C. jejuni*, resveratrol exerted direct barrier-improving effects by preventing enhanced epithelial apoptosis and restoring TJ dysregulated claudins into the TJ domain. Furthermore, we speculate that epithelial defense mechanisms may be enhanced by resveratrol. In general, the mucosal defense against microorganisms can be modulated by increased expression of mucins, tight junctional proteins, secretion of chemokines and cytokines, or release of direct antimicrobials like *β*-defensins and cathelicidin. We know from other studies that resveratrol affects intracellular signaling pathways, for example, blockage of NFκB activation (Singh et al., 2010), activator protein-1 (Kundu and Surh, 2004), IκBα (Tsai et al., 1999) or TLR4 and STAT3 (Zhang et al., 2019). Notably, a recent study showed the synergistic effect of resveratrol and vitamin D on the heterodimerization of VDR-RXR nuclear factors, which reflected an increase in vitamin D-dependent gene expression involving transactivation by resveratrol (Dampf Stone et al., 2015). We have previously shown this vitamin D-dependent pathway to be affected in campylobacteriosis and provided the first evidence that supplementation with active vitamin D antagonized the effects of *C. jejuni in vitro* and to some extent *in vivo* (Bücker et al., 2018; Mousavi et al., 2019). It would therefore seem reasonable that novel therapeutic approaches should involve a combination of compounds, in order to use their synergistic effects to increase their overall efficacy. Agents that inhibit transepithelial migration of *C. jejuni* may also be valuable. And it is interesting that resveratrol reduced parasite translocation in a mouse model of intestinal *Toxoplasma gondii* infection (Bereswill et al., 2010). Thus, a barrier-improving agent such as resveratrol could be combined with an immune modulator such as curcumin or vitamin D.

In conclusion, the effects of *C. jejuni* infection can be attenuated by agents with barrier-protective, anti-inflammatory or anti-apoptotic actions, alone or in combination. Promising compounds to treat or prevent *Campylobacter* infections are nutraceuticals such as resveratrol or curcumin, which should be considered as therapeutic options in multimodal approaches to treat acute enteritis or post-infective IBS. Resveratrol is particularly effective in restoring the epithelial leak pathway and may prevent the pathogenesis of a *leaky gut*.

## Data Availability

The raw data supporting the conclusions of this article will be made available by the authors, without undue reservation.

## References

[B1] Al-Salloom F. Mahmeed A. A. Ismaeel A. Botta G. A. Bakhiet M. (2003). *Campylobacter*-stimulated INT407 cells produce dissociated cytokine profiles. J. Infect. 47 (3), 217–224. 10.1016/s0163-4453(03)00076-8 12963383

[B2] Amasheh M. Fromm A. Krug S. M. Amasheh S. Andres S. Zeitz M. (2010). TNFα-induced and berberine-antagonized tight junction barrier impairment via tyrosine kinase, Akt and NFκB signaling. J. Cell Sci. 123 (23), 4145–4155. 10.1242/jcs.070896 21062898

[B3] Bereswill S. Muñoz M. Fischer A. Plickert R. Haag L.-M. Otto B. (2010). Anti-inflammatory effects of resveratrol, curcumin and simvastatin in acute small intestinal inflammation. PLoS One 5 (12), e15099. 10.1371/journal.pone.0015099 21151942 PMC2997083

[B4] Bergann T. Plöger S. Fromm A. Zeissig S. Borden S. A. Fromm M. (2009). A colonic mineralocorticoid receptor cell model expressing epithelial Na+ channels. Biochem. Biophys. Res. Comm. 381 (2), 280–285. 10.1016/j.bbrc.2009.03.006 19275887

[B5] Berman A. Y. Motechin R. A. Wiesenfeld M. Y. Holz M. K. (2017). The therapeutic potential of resveratrol: a review of clinical trials. npj Precision Onc 1, 35. 10.1038/s41698-017-0038-6 PMC5630227 28989978

[B6] Black R. E. Levine M. M. Clements M. L. Hughes T. P. Blaser M. J. (1988). Experimental *Campylobacter jejuni* infection in humans. J. Infect. Dis. 157 (3), 472–479. 10.1093/infdis/157.3.472 3343522

[B7] Bojarski C. Weiske J. Schöneberg T. Schröder W. Mankertz J. Schulzke J. D. (2004). The specific fates of tight junction proteins in apoptotic epithelial cells. J. Cell Sci. 117 (10), 2097–2107. 10.1242/jcs.01071 15054114

[B8] Bücker R. Krug S. M. Fromm A. Nielsen H. L. Fromm M. Nielsen H. (2017). *Campylobacter* fetusimpairs barrier function in HT-29/B6 cells through focal tight junction alterations and leaks. Ann. N.Y. Acad. Sci. 1405 (1), 189–201. 10.1111/nyas.13406 28662272

[B9] Bücker R. Krug S. M. Moos V. Bojarski C. Schweiger M. R. Kerick M. (2018). Erratum: *Campylobacter* jejuni impairs sodium transport and epithelial barrier function via cytokine release in human colon. Mucosal Immunol. 11 (2), 575–577. 10.1038/mi.2017.78 29091080

[B10] Bücker R. Schulz E. Günzel D. Bojarski C. Lee I.-F. M. John L. J. (2014). α-Haemolysin ofEscherichia coliin IBD: a potentiator of inflammatory activity in the colon. Gut 63 (12), 1893–1901. 10.1136/gutjnl-2013-306099 24534723

[B11] Butkevych E. Lobo de Sá F. D. Nattramilarasu P. K. Bücker R. (2020). Contribution of epithelial apoptosis and subepithelial immune responses in *Campylobacter jejuni*-induced barrier disruption. Front. Microbiol. 11, 344. 10.3389/fmicb.2020.00344 32210941 PMC7067706

[B12] Cao S. Shen Z. Wang C. Zhang Q. Hong Q. He Y. (2019). Resveratrol improves intestinal barrier function, alleviates mitochondrial dysfunction and induces mitophagy in diquat challenged piglets1. Food Funct. 10 (1), 344–354. 10.1039/c8fo02091d 30601541

[B13] Carrasco-Pozo C. Morales P. Gotteland M. (2013). Polyphenols protect the epithelial barrier function of Caco-2 cells exposed to indomethacin through the modulation of occludin and zonula occludens-1 expression. J. Agric. Food Chem. 61 (22), 5291–5297. 10.1021/jf400150p 23668856

[B14] Chen M. L. Ge Z. Fox J. G. Schauer D. B. (2006). Disruption of tight junctions and induction of proinflammatory cytokine responses in colonic epithelial cells by *Campylobacter jejuni* . Iai 74 (12), 6581–6589. 10.1128/IAI.00958-06 PMC1698078 17015453

[B15] Chudzińska M. Rogowicz D. Wołowiec Ł. Banach J. Sielski S. Bujak R. (2020). Resveratrol and cardiovascular system-the unfulfilled hopes. Ir J. Med. Sci. 10.1007/s11845-020-02441-x 33219913

[B16] Dampf Stone A. Batie S. F. Sabir M. S. Jacobs E. T. Lee J. H. Whitfield G. K. (2015). Resveratrol potentiates vitamin D and nuclear receptor signaling. J. Cell. Biochem. 116 (6), 1130–1143. 10.1002/jcb.25070 25536521

[B17] Edwards L. A. Nistala K. Mills D. C. Stephenson H. N. Zilbauer M. Wren B. W. (2010). Delineation of the innate and adaptive T-cell immune outcome in the human host in response to *Campylobacter jejuni* infection. PLoS One 5 (11), e15398. 10.1371/journal.pone.0015398 21085698 PMC2976761

[B18] El-Kott A. F. Shati A. A. Ali Al-Kahtani M. Alharbi S. A. (2019). The apoptotic effect of resveratrol in ovarian cancer cells is associated with downregulation of galectin-3 and stimulating miR-424-3p transcription. J. Food Biochem. 43 (12), e13072. 10.1111/jfbc.13072 31603261

[B19] Feng L. Yasmeen R. Schoene N. W. Lei K. Y. Wang T. T. Y. (2019). Resveratrol differentially modulates immune responses in human THP-1 monocytes and macrophages. Nutr. Res. 72, 57–69. 10.1016/j.nutres.2019.10.003 31757634

[B20] Gao X. Xu Y. X. Janakiraman N. Chapman R. A. Gautam∗ S. C. (2001). Immunomodulatory activity of resveratrol: suppression of lymphocyte proliferation, development of cell-mediated cytotoxicity, and cytokine production11Abbreviations: CTLs, cytotoxic T lymphocytes; LAK cells, lymphokine activated killer cells; IL-2, interleukin-2; IFN-γ, interferon-gamma; TNF-α, tumor necrosis factor-α NF-κB, nuclear factor kappa B; Con A, concanavalin A; HBSS, Hanks' balanced salt solution; DTT, dithiothreitol; PMSF, phenylmethylsulfonyl fluoride; RT-PCR, reverse transcription-polymerase chain reaction; LPS, lipopolysaccharide; and EMSA, electrophoretic mobility shift assay. Biochem. Pharmacol. 62, 1299–1308. 10.1016/s0006-2952(01)00775-4 11705464

[B21] Gölz G. Karadas G. Alutis M. E. Fischer A. Kühl A. A. Breithaupt A. (2015). *Arcobacter butzleri* induce colonic, extra-intestinal and systemic inflammatory responses in Gnotobiotic IL-10 deficient mice in a strain-dependent manner. PLoS One 10 (9), e0139402. 10.1371/journal.pone.0139402 26406497 PMC4584000

[B22] Haag L.-M. Fischer A. Otto B. Plickert R. Kühl A. A. Göbel U. B. (2012). *Campylobacter* jejuni induces acute enterocolitis in Gnotobiotic IL-10−/− mice via toll-like-receptor-2 and -4 signaling. PLoS One 7 (7), e40761. 10.1371/journal.pone.0040761 22808254 PMC3393706

[B23] Harrer A. Bücker R. Boehm M. Zarzecka U. Tegtmeyer N. Sticht H. (2019). *Campylobacter jejuni* enters gut epithelial cells and impairs intestinal barrier function through cleavage of occludin by serine protease HtrA. Gut Pathog. 11, 4. 10.1186/s13099-019-0283-z 30805031 PMC6373145

[B24] Heimesaat M. M. Escher U. Grunau A. Kühl A. A. Bereswill S. (2019). Multidrug-resistant *Pseudomonas aeruginosa* accelerate intestinal, extra-intestinal, and systemic inflammatory responses in human microbiota-associated mice with subacute ileitis. Front. Immunol. 10, 49. 10.3389/fimmu.2019.00049 30761129 PMC6361842

[B25] Heimesaat M. M. Mousavi S. Escher U. Lobo de Sá F. D. Peh E. Schulzke J.-D. (2020). Resveratrol alleviates acute *Campylobacter* jejuni induced enterocolitis in a preclinical murine intervention study. Microorganisms 8 (12), 1858. 10.3390/microorganisms8121858 PMC7760181 33255723

[B26] Hollander D. (1999). Intestinal permeability, leaky gut, and intestinal disorders. Curr. Gastroenterol. Rep. 1 (5), 410–416. 10.1007/s11894-999-0023-5 10980980

[B27] Klančnik A. Šikić Pogačar M. Trošt K. Tušek Žnidarič M. Mozetič Vodopivec B. Smole Možina S. (2017). Anti-*Campylobacter* activity of resveratrol and an extract from waste Pinot noir grape skins and seeds, and resistance of *Camp. jejuni* planktonic and biofilm cells, mediated via the CmeABC efflux pump. J. Appl. Microbiol. 122 (1), 65–77. 10.1111/jam.13315 27709726

[B28] Koushki M. Amiri-Dashatan N. Ahmadi N. Abbaszadeh H. A. Rezaei-Tavirani M. (2018a). Resveratrol: a miraculous natural compound for diseases treatment. Food Sci. Nutr. 6 (8), 2473–2490. 10.1002/fsn3.855 30510749 PMC6261232

[B29] Koushki M. Dashatan N. A. Meshkani R. (2018b). Effect of resveratrol supplementation on inflammatory markers: a systematic review and meta-analysis of randomized controlled trials. Clin. Ther. 40 (7), 1180–1192. 10.1016/j.clinthera.2018.05.015 30017172

[B30] Kundu J. K. Surh Y.-J. (2004). Molecular basis of chemoprevention by resveratrol: NF-κB and AP-1 as potential targets. Mutat. Research/Fundamental Mol. Mech. Mutagenesis 555 (1-2), 65–80. 10.1016/j.mrfmmm.2004.05.019 15476852

[B31] Ling K.-H. Wan M. L. Y. El-Nezami H. Wang M. (2016). Protective capacity of resveratrol, a natural polyphenolic compound, against deoxynivalenol-induced intestinal barrier dysfunction and bacterial translocation. Chem. Res. Toxicol. 29 (5), 823–833. 10.1021/acs.chemrestox.6b00001 27058607

[B32] Liu J. Zhang X. Yan T. Wang F. Li J. Jia L. (2020). Screening of an endophyte transforming polydatin to resveratrol from *Reynoutria japonica* houtt and the optimization of its transformation parameters. Molecules 25 (20), 4830. 10.3390/molecules25204830 PMC7587952 33092209

[B60] Lobo de Sá F. D. Butkevych E. Nattramilarasu P. K. Fromm A. Mousavi S. Moos V. (2019). Curcumin mitigates immune-induced epithelial barrier dysfunction by Campylobacter jejuni. Int. J. Mol. Sci. 20 (19), 4830. 10.3390/ijms20194830 PMC6802366 31569415

[B59] Lobo de Sá F. D. Schulzke J. D. Bűcker R. (2021). Diarrheal mechanisms and the role of intestinal barrier dysfunction in Campylobacter infections. Curr. Top. Microbiol. Immunol. 431, 203–231. 10.1007/978-3-030-65481-8_8 33620653

[B33] Luettig J. Rosenthal R. Lee I.-F. M. Krug S. M. Schulzke J. D. (2016). The ginger component 6-shogaol prevents TNF-α-induced barrier loss via inhibition of PI3K/Akt and NF-κB signaling. Mol. Nutr. Food Res. 60 (12), 2576–2586. 10.1002/mnfr.201600274 27487982

[B34] Mayangsari Y. Suzuki T. (2018). Resveratrol ameliorates intestinal barrier defects and inflammation in colitic mice and intestinal cells. J. Agric. Food Chem. 66 (48), 12666–12674. 10.1021/acs.jafc.8b04138 30426751

[B35] Mousavi S. Bereswill S. Heimesaat M. (2020). Novel clinical *Campylobacter jejuni* infection models based on sensitization of mice to lipooligosaccharide, a major bacterial factor triggering innate immune responses in human campylobacteriosis. Microorganisms 8 (4), 482. 10.3390/microorganisms8040482 PMC7232424 32231139

[B36] Mousavi S. Lobo de Sá F. D. Schulzke J.-D. Bücker R. Bereswill S. Heimesaat M. M. (2019). Vitamin D in acute campylobacteriosis-results from an intervention study applying a clinical *Campylobacter jejuni* induced enterocolitis model. Front. Immunol. 10, 2094. 10.3389/fimmu.2019.02094 31552040 PMC6735268

[B37] Nielsen H. L. Engberg J. Ejlertsen T. Bücker R. Nielsen H. (2012). Short-term and medium-term clinical outcomes of *Campylobacter concisus* infection. Clin. Microbiol. Infect. 18 (11), E459–E465. 10.1111/j.1469-0691.2012.03990.x 22882347

[B38] Otto B. Haag L.-M. Fischer A. Plickert R. Kühl A. A. Göbel U. B. (2012). *Campylobacter* jejuniinduces extra-intestinal immune responses via toll-like-receptor-4 signaling in conventional IL-10 deficient mice with chronic colitis. Eur. J. Microbiol. Immunol. (Bp) 2 (3), 210–219. 10.1556/EuJMI.2.2012.3.7 24688768 PMC3962757

[B39] Rees L. E. N. Cogan T. A. Dodson A. L. Birchall M. A. Bailey M. Humphrey T. J. (2008). *Campylobacter* and IFNγ interact to cause a rapid loss of epithelial barrier integrity. Inflamm. Bowel Dis. 14 (3), 303–309. 10.1002/ibd.20325 18050297

[B40] Rosenthal R. Luettig J. Hering N. A. Krug S. M. Albrecht U. Fromm M. (2017). Myrrh exerts barrier-stabilising and -protective effects in HT-29/B6 and Caco-2 intestinal epithelial cells. Int. J. Colorectal Dis. 32 (5), 623–634. 10.1007/s00384-016-2736-x 27981377

[B41] Salehi B. Mishra A. Nigam M. Sener B. Kilic M. Sharifi-Rad M. (2018). Resveratrol: a double-edged sword in health benefits. Biomedicines 6 (3), 91. 10.3390/biomedicines6030091 PMC6164842 30205595

[B42] Schulzke J.-D. Bojarski C. Zeissig S. Heller F. Gitter A. H. Fromm M. (2006). Disrupted barrier function through epithelial cell apoptosis. Ann. New York Acad. Sci. 1072, 288–299. 10.1196/annals.1326.027 17057208

[B43] Shane S. M. (1992). The significance ofcampylobacter jejuniinfection in poultry: a review. Avian Pathol. 21 (2), 189–213. 10.1080/03079459208418836 18670933

[B44] Singh S. K. Banerjee S. Acosta E. P. Lillard J. W. Singh R. (2017). Resveratrol induces cell cycle arrest and apoptosis with docetaxel in prostate cancer cells via a p53/p21WAF1/CIP1 and p27KIP1 pathway. Oncotarget 8 (10), 17216–17228. 10.18632/oncotarget.15303 28212547 PMC5370034

[B45] Singh U. P. Singh N. P. Singh B. Hofseth L. J. Price R. L. Nagarkatti M. (2010). Resveratrol (Trans-3,5,4′-trihydroxystilbene) induces silent mating type information regulation-1 and down-regulates nuclear transcription factor-κb activation to abrogate dextran sulfate sodium-induced colitis. J. Pharmacol. Exp. Ther. 332 (3), 829–839. 10.1124/jpet.109.160838 19940103 PMC2835444

[B46] Stahl M. Ries J. Vermeulen J. Yang H. Sham H. P. Crowley S. M. (2014). A novel mouse model of *Campylobacter* jejuni gastroenteritis reveals key pro-inflammatory and tissue protective roles for Toll-like receptor signaling during infection. Plos Pathog. 10 (7), e1004264. 10.1371/journal.ppat.1004264 25033044 PMC4102570

[B47] Stahl M. Vallance B. A. (2015). Insights intoCampylobacter jejunicolonization of the mammalian intestinal tract using a novel mouse model of infection. Gut Microbes 6 (2), 143–148. 10.1080/19490976.2015.1016691 25831043 PMC4615362

[B48] Tsai S. H. Lin-Shiau S. Y. Lin J. K. (1999). Suppression of nitric oxide synthase and the down-regulation of the activation of NFκB in macrophages by resveratrol. Br. J. Pharmacol. 126 (3), 673–680. 10.1038/sj.bjp.0702357 10188978 PMC1565862

[B49] Vandamme P. Falsen E. Rossau R. Hoste B. Segers P. Tytgat R. (1991). Revision of *Campylobacter, Helicobacter*, and *Wolinella* taxonomy: emendation of generic descriptions and proposal of *Arcobacter* gen. nov. Int. J. Syst. Bacteriol. 41 (1), 88–103. 10.1099/00207713-41-1-88 1704793

[B50] von Klitzing E. Ekmekciu I. Kühl A. A. Bereswill S. Heimesaat M. M. (2017). Intestinal, extra-intestinal and systemic sequelae of *Toxoplasma gondii* induced acute ileitis in mice harboring a human gut microbiota. PLoS One 12 (4), e0176144. 10.1371/journal.pone.0176144 28414794 PMC5393883

[B51] Wang N. Han Q. Wang G. Ma W. P. Wang J. Wu W. X. (2016). Resveratrol protects oxidative stress-induced intestinal epithelial barrier dysfunction by upregulating heme oxygenase-1 expression. Dig. Dis. Sci. 61 (9), 2522–2534. 10.1007/s10620-016-4184-4 27146412

[B52] Wassenaar T. M. Blaser M. J. (1999). Pathophysiology of *Campylobacter jejuni* infections of humans. Microbes Infect. 1 (12), 1023–1033. 10.1016/s1286-4579(99)80520-6 10617934

[B53] Weiskirchen S. Weiskirchen R. (2016). Resveratrol: how much wine do you have to drink to stay healthy?. Adv. Nutr. 7 (4), 706–718. 10.3945/an.115.011627 27422505 PMC4942868

[B54] Yao J. Wang J. Y. Liu L. Li Y. X. Xun A. Y. Zeng W. S. (2010). Anti-oxidant effects of resveratrol on mice with DSS-induced ulcerative colitis. Arch. Med. Res. 41 (4), 288–294. 10.1016/j.arcmed.2010.05.002 20637373

[B55] Yao J. Wei C. Wang J. Y. Zhang R. Li Y. X. Wang L. S. (2015). Effect of resveratrol on Treg/Th17 signaling and ulcerative colitis treatment in mice. Wjg 21 (21), 6572–6581. 10.3748/wjg.v21.i21.6572 26074695 PMC4458767

[B56] Zhang M. Xue Y. Chen H. Meng L. Chen B. Gong H. (2019). Resveratrol inhibits MMP3 and MMP9 expression and secretion by suppressing TLR4/NF-κB/STAT3 activation in ox-LDL-treated HUVECs. Oxid Med. Cell Longev, 1. 10.1155/2019/9013169 PMC6754947 31583048

[B57] Zhao L. Lee J. Y. Hwang D. H. (2011). Inhibition of pattern recognition receptor-mediated inflammation by bioactive phytochemicals. Nutr. Rev. 69 (6), 310–320. 10.1111/j.1753-4887.2011.00394.x 21631512 PMC3881972

[B58] Zhong M. Cheng G. F. Wang W. J. Guo Y. Zhu X. Y. Zhang J. T. (1999). Inhibitory effect of resveratrol on interleukin 6 release by stimulated peritoneal macrophages of mice. Phytomedicine 6 (2), 79–84. 10.1016/S0944-7113(99)80039-7 10374244

